# Supplementary material for: Optimized Bioproduction of Itaconic and Fumaric Acids Based on Solid-State Fermentation of Lignocellulosic Biomass
Source: Molecules. 2020 Feb 27;25(5):1070. doi: 10.3390/molecules25051070 (PMC7179149; doi:10.3390/molecules25051070)
Supplement: Supplementary file 1 [file molecules-25-01070-s001.pdf]

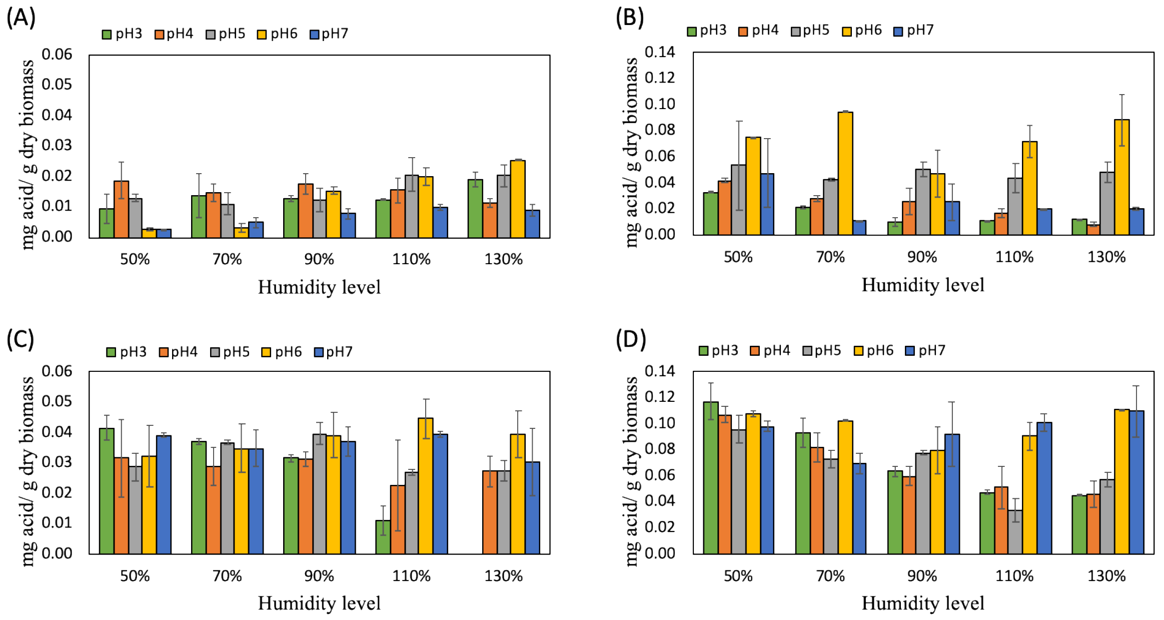

Figure S1. SSF on corn cobs at different pH and moisture levels by *A. terreus* (IA and FA yields: **A** and **B**, respectively) and *A. oryzae* (**C** and **D**, respectively).

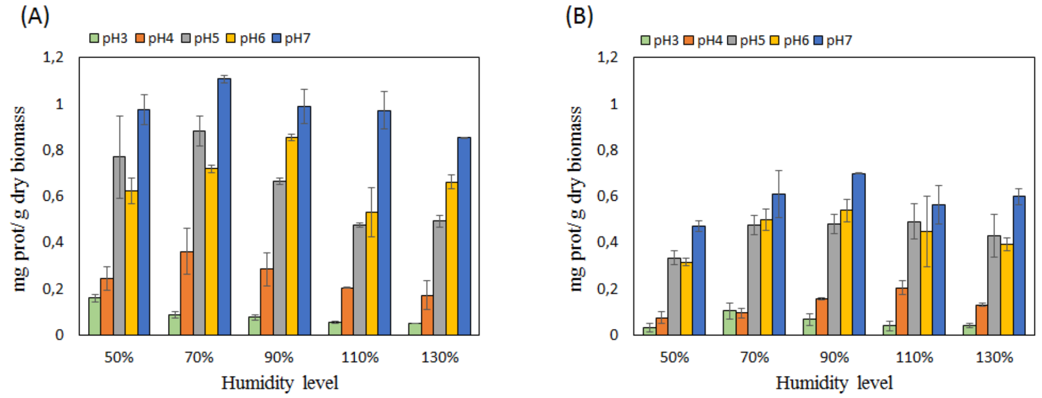

Figure S2. Fungal growth (proteins productions) under different pH and moisture conditions. (**A**) SSF of corn cobs by *A. terreus* and (**B**) SSF of corn cobs by *A. oryzae*.
